# Supplementary material for: Cross-linking of T cell to B cell lymphoma by the T cell bispecific antibody CD20-TCB induces IFNγ/CXCL10-dependent peripheral T cell recruitment in humanized murine model
Source: PLoS One. 2021 Jan 6;16(1):e0241091. doi: 10.1371/journal.pone.0241091 (PMC7787458; doi:10.1371/journal.pone.0241091)
Supplement: S1 Fig — a) Schematic of CD20-TCB structure. In blue are indicated two high affinity binding sites for CD20, leading to tumor cell targeting. In orange the high affinity binding site for CD3, leading to T cell engagement. In gray the heterodimeric human IgG1 carrying the “PG LALA” mutations b) schematic showing CD20-TCB targeting CD20 on tumor cells and CD3 on T cells, leading to tumor recognition, by conventional T cells, in an antigen-independent manner (TCR-independent). Adapted from https://smart.servier.com/ c) Viability assay of OCI-Ly18 cells by AnnexinV (AnnV+) and Propidium Iodide (PI+) staining, 16 hours after co-culture with CD3/CD28 activated CD8+ T cells treated at the indicated doses of CD20-TCB; +/- LFA1 inhibitor (10 μg/ml). n = 3 per point. Mean and +/- s.d. are shown. 2way-Anova, * p<0.05. d) Viability assay of CD8+ by Ann V and PI staining, 16 hours after co-culture with WSU DLCL2 or OCI-Ly18 target cells treated at the indicated doses of CD20-TCB; +/- LFA1 inhibitor (10 μg/ml). n = 4 per point. Mean and +/- s.d. are shown. e) Representative confocal imaging of LFA1 (white) localization at the synapse between T cell (F-actin is shown in green) and target cell (blue) 4 hours after CD20-TCB treatment with (bottom) or without (top) LFA1 inhibitor (10 μg/ml) treatment. f) Flow cytometry analysis of CD20 and ICAM-1 expression of cell surface of WSU DLCL2 and OCI-Ly18 cells. g) Top: CD20 mean fluorescent intensity (MFI) and bottom: ICAM-1 MFI. The MFI has been correlated to the percentage of killed cells at high dose of CD20-TCB (200 ng/ml) on indicated DLCBL cell lines, as assessed by flow cytometry. (PPTX) [file pone.0241091.s001.pptx]

## Slide 1
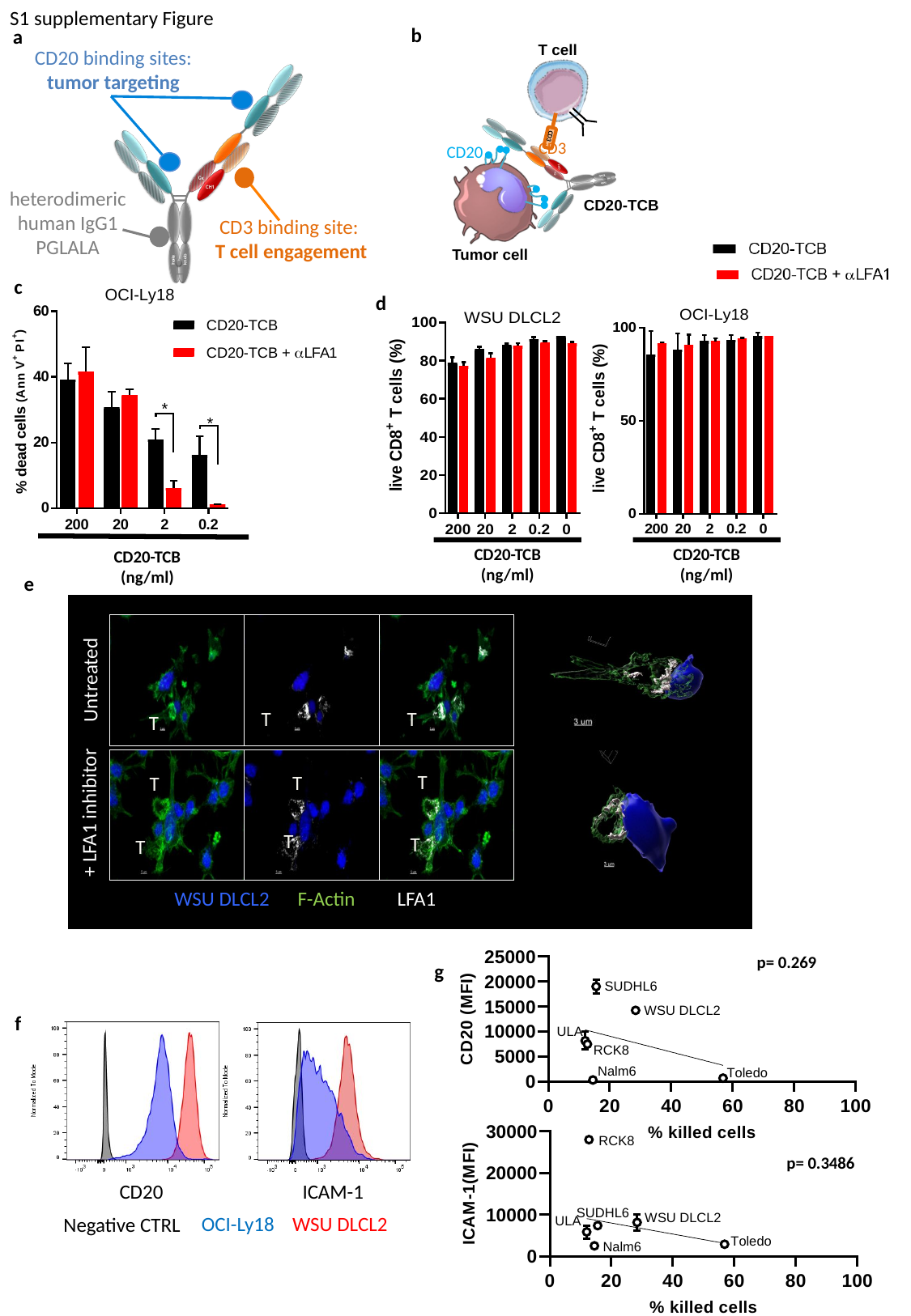

S1 supplementary Figure
b
a
T cell
CD3
CD20
Tumor cell
CD20-TCB
CD20 binding sites: tumor targeting
CD3
heterodimeric human IgG1 PGLALA
CD3 binding site:
T cell engagement
d
CD20-TCB
(ng/ml)
CD20-TCB
(ng/ml)
c
CD20-TCB
(ng/ml)
Untreated
T
T
T
T
T
T
T
T
T
WSU DLCL2 F-Actin LFA1
+ LFA1 inhibitor
e
p= 0.269
g
f
ICAM-1
CD20
OCI-Ly18
WSU DLCL2
Negative CTRL
p= 0.3486

## Slide 2
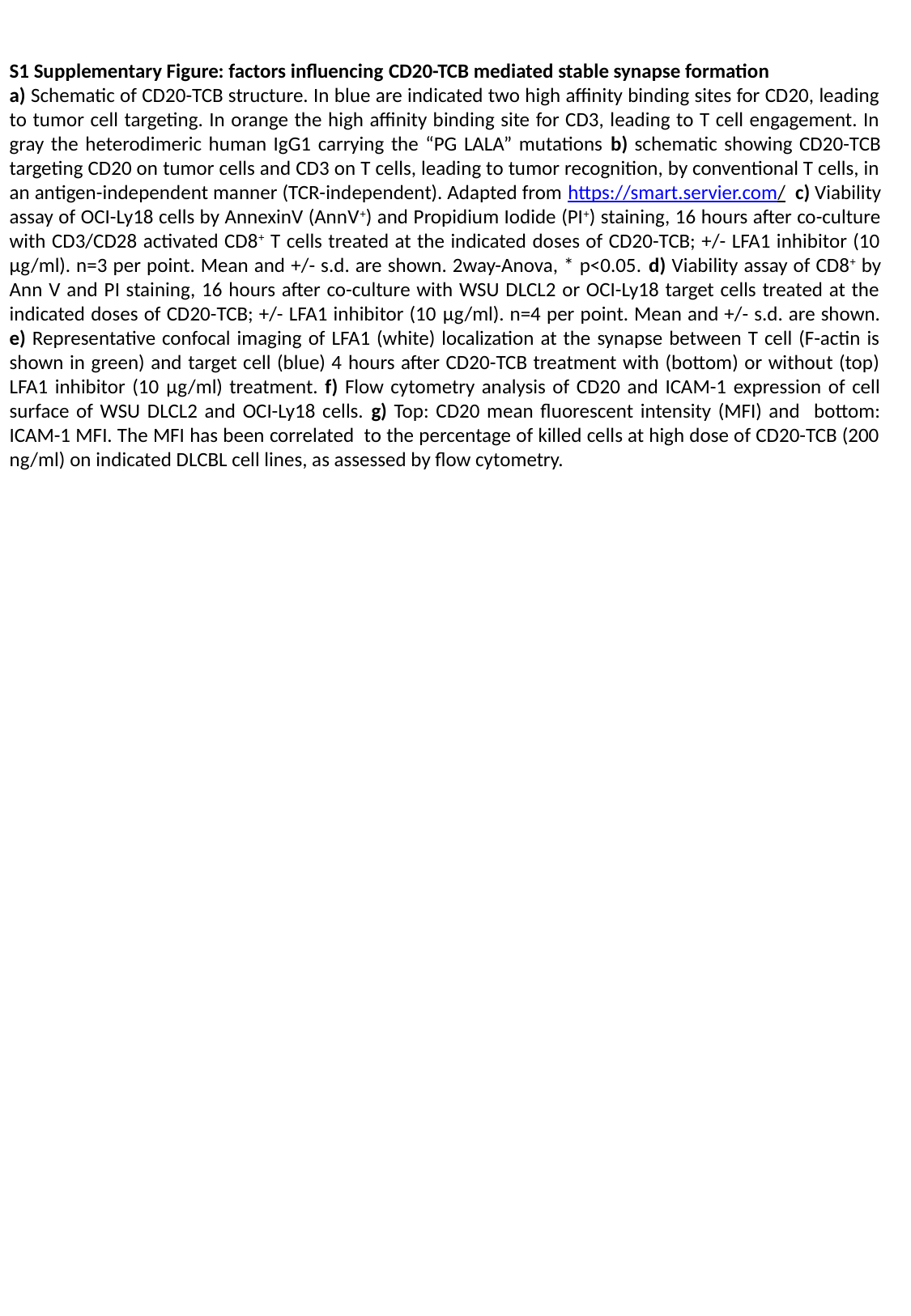

S1 Supplementary Figure: factors influencing CD20-TCB mediated stable synapse formation
a) Schematic of CD20-TCB structure. In blue are indicated two high affinity binding sites for CD20, leading to tumor cell targeting. In orange the high affinity binding site for CD3, leading to T cell engagement. In gray the heterodimeric human IgG1 carrying the “PG LALA” mutations b) schematic showing CD20-TCB targeting CD20 on tumor cells and CD3 on T cells, leading to tumor recognition, by conventional T cells, in an antigen-independent manner (TCR-independent). Adapted from https://smart.servier.com/ c) Viability assay of OCI-Ly18 cells by AnnexinV (AnnV+) and Propidium Iodide (PI+) staining, 16 hours after co-culture with CD3/CD28 activated CD8+ T cells treated at the indicated doses of CD20-TCB; +/- LFA1 inhibitor (10 μg/ml). n=3 per point. Mean and +/- s.d. are shown. 2way-Anova, * p<0.05. d) Viability assay of CD8+ by Ann V and PI staining, 16 hours after co-culture with WSU DLCL2 or OCI-Ly18 target cells treated at the indicated doses of CD20-TCB; +/- LFA1 inhibitor (10 μg/ml). n=4 per point. Mean and +/- s.d. are shown. e) Representative confocal imaging of LFA1 (white) localization at the synapse between T cell (F-actin is shown in green) and target cell (blue) 4 hours after CD20-TCB treatment with (bottom) or without (top) LFA1 inhibitor (10 μg/ml) treatment. f) Flow cytometry analysis of CD20 and ICAM-1 expression of cell surface of WSU DLCL2 and OCI-Ly18 cells. g) Top: CD20 mean fluorescent intensity (MFI) and bottom: ICAM-1 MFI. The MFI has been correlated to the percentage of killed cells at high dose of CD20-TCB (200 ng/ml) on indicated DLCBL cell lines, as assessed by flow cytometry.
