# Supplementary material for: Cross-linking of T cell to B cell lymphoma by the T cell bispecific antibody CD20-TCB induces IFNγ/CXCL10-dependent peripheral T cell recruitment in humanized murine model
Source: PLoS One. 2021 Jan 6;16(1):e0241091. doi: 10.1371/journal.pone.0241091 (PMC7787458; doi:10.1371/journal.pone.0241091)
Supplement: S2 Fig — a) Representative MP-IVM imaging on HSC-NSG-NSG mice showing localization of therapy 2 hours after intradermal treatment with 0.5 mg/kg CD20-TCB. Blue: WSU DLCL2 tumor cells; Pink: T cells; White: Labeled CD20-TCB. The yellow arrows indicate the therapy polarization points at the T cells-tumor cells contact sites. b-c) Percentage of (b) Ki67+ and (c) Granzyme B+ cells among huCD45+ CD3+ CD8+ cells from the blood of PBMC-NSG vs HSC-NSG mice. n = 5 per group, mean +/- s.d. are shown. Unpaired t-test, ****p<0.0001. c) Percentage of naïve CD3+ cells (CD62L+,CD45RA+) among huCD45+ from the blood of PBMC-NSG mice vs HSC-NSG mice. n = 3 per group, mean +/- s.d. are shown. Unpaired t-test, ****p<0.0001. (PPTX) [file pone.0241091.s002.pptx]

## Slide 1
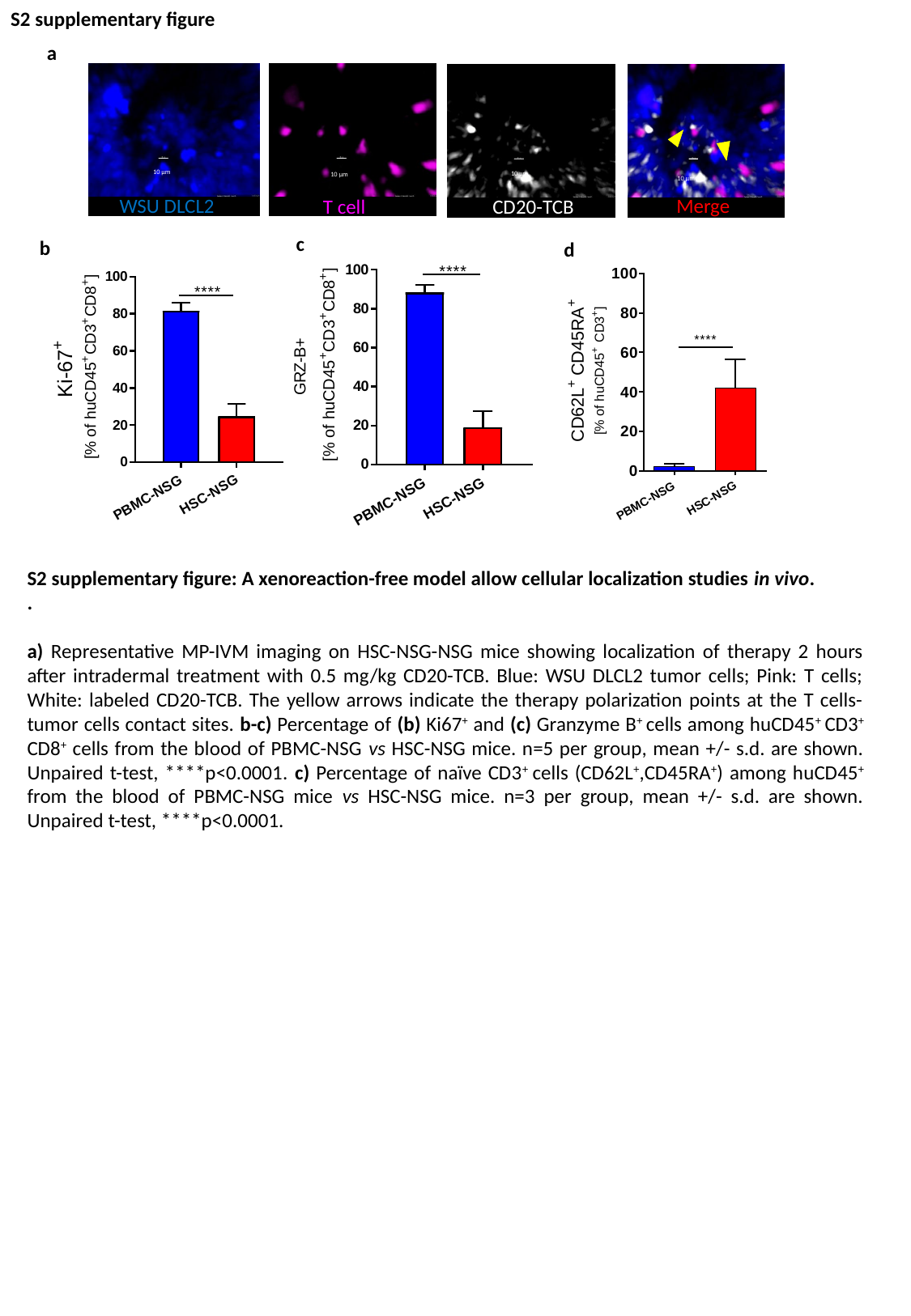

S2 supplementary figure
a
10 μm
10 μm
10 μm
10 μm
Merge
WSU DLCL2
T cell
CD20-TCB
b
c
d
S2 supplementary figure: A xenoreaction-free model allow cellular localization studies in vivo.
.
a) Representative MP-IVM imaging on HSC-NSG-NSG mice showing localization of therapy 2 hours after intradermal treatment with 0.5 mg/kg CD20-TCB. Blue: WSU DLCL2 tumor cells; Pink: T cells; White: labeled CD20-TCB. The yellow arrows indicate the therapy polarization points at the T cells-tumor cells contact sites. b-c) Percentage of (b) Ki67+ and (c) Granzyme B+ cells among huCD45+ CD3+ CD8+ cells from the blood of PBMC-NSG vs HSC-NSG mice. n=5 per group, mean +/- s.d. are shown. Unpaired t-test, ****p<0.0001. c) Percentage of naïve CD3+ cells (CD62L+,CD45RA+) among huCD45+ from the blood of PBMC-NSG mice vs HSC-NSG mice. n=3 per group, mean +/- s.d. are shown. Unpaired t-test, ****p<0.0001.
10 μm
