# Supplementary material for: Cross-linking of T cell to B cell lymphoma by the T cell bispecific antibody CD20-TCB induces IFNγ/CXCL10-dependent peripheral T cell recruitment in humanized murine model
Source: PLoS One. 2021 Jan 6;16(1):e0241091. doi: 10.1371/journal.pone.0241091 (PMC7787458; doi:10.1371/journal.pone.0241091)
Supplement: S3 Fig — a) Percentage of Granzyme B+ CD8 + and (b) Perforin+ CD8 + T cells at 24h, 48h and 72h post CD20-TCB stimulation in vitro, as assessed by flow cytometry. CD8+ T cells were freshly purified from PBMCs and stimulated with 200 ng/ml of CD20-TCB in the presence of WSU DLCL2 as target cells. n = 3 per group, mean and s.d. are shown. Two-way Anova ***p<0.005, ****p<0.0001. a) Percentage of Granzyme B+ CD8+ and (b) Perforin+ CD8+ T cells at 24h, 48h and 72h post CD20-TCB stimulation in vitro, as assessed by flow cytometry. CD8+ T cells were freshly purified from PBMCs and stimulated with 200 ng/ml of CD20-TCB in the presence of WSU DLCL2 as target cells. n = 3 per group, mean and s.d. are shown. Two-way Anova ***p<0.005, ****p<0.0001. (PPTX) [file pone.0241091.s003.pptx]

## Slide 1
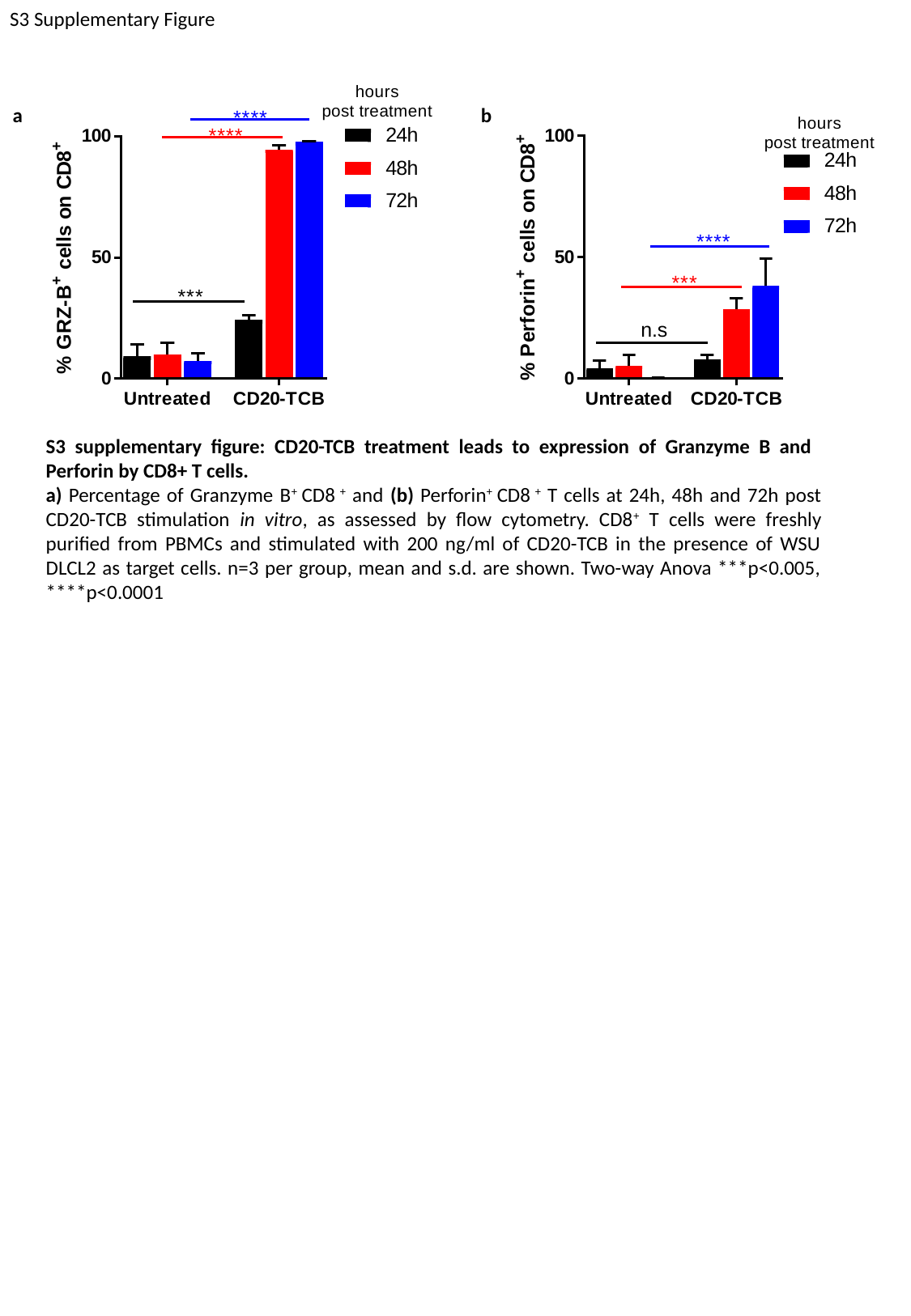

S3 Supplementary Figure
a
b
S3 supplementary figure: CD20-TCB treatment leads to expression of Granzyme B and Perforin by CD8+ T cells.
a) Percentage of Granzyme B+ CD8 + and (b) Perforin+ CD8 + T cells at 24h, 48h and 72h post CD20-TCB stimulation in vitro, as assessed by flow cytometry. CD8+ T cells were freshly purified from PBMCs and stimulated with 200 ng/ml of CD20-TCB in the presence of WSU DLCL2 as target cells. n=3 per group, mean and s.d. are shown. Two-way Anova ***p<0.005, ****p<0.0001
