# Supplementary material for: Cross-linking of T cell to B cell lymphoma by the T cell bispecific antibody CD20-TCB induces IFNγ/CXCL10-dependent peripheral T cell recruitment in humanized murine model
Source: PLoS One. 2021 Jan 6;16(1):e0241091. doi: 10.1371/journal.pone.0241091 (PMC7787458; doi:10.1371/journal.pone.0241091)
Supplement: S4 Fig — a) Percentage of CD69+ CD8+ (left) and CD25+ CD8+ (right) T cells at 24h, 48h and 72h post CD20-TCB stimulation in vitro, as assessed by flow cytometry. PBMCs-freshly purified CD8+ T cells were stimulated at the indicated doses of CD20-TCB in the presence of WSU DLCL2 as target cells. n = 3 per group, mean and s.d. are shown. b) Speed, Track displacement and Arrest coefficient of resident T cells and recruited cells at 72h post treatment. Shown are individual mean values per movie, Mean +/- s.d. Statistics: Unpaired 2-tailed t-test with Welch’s correction. *** p<0.005; **** p< 0.0001. (PPTX) [file pone.0241091.s004.pptx]

## Slide 1
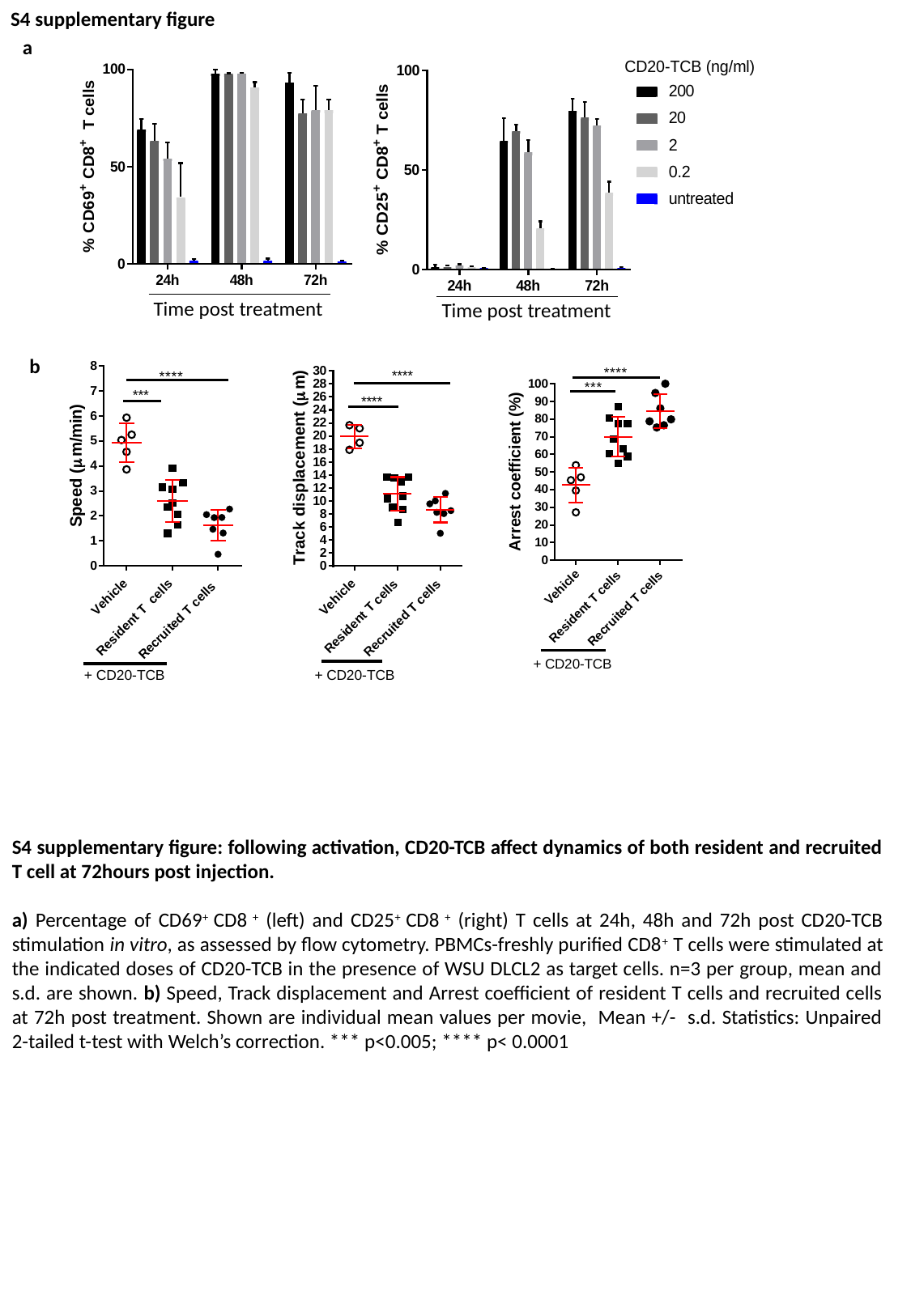

S4 supplementary figure
a
Time post treatment
Time post treatment
b
S4 supplementary figure: following activation, CD20-TCB affect dynamics of both resident and recruited T cell at 72hours post injection.
a) Percentage of CD69+ CD8 + (left) and CD25+ CD8 + (right) T cells at 24h, 48h and 72h post CD20-TCB stimulation in vitro, as assessed by flow cytometry. PBMCs-freshly purified CD8+ T cells were stimulated at the indicated doses of CD20-TCB in the presence of WSU DLCL2 as target cells. n=3 per group, mean and s.d. are shown. b) Speed, Track displacement and Arrest coefficient of resident T cells and recruited cells at 72h post treatment. Shown are individual mean values per movie, Mean +/- s.d. Statistics: Unpaired 2-tailed t-test with Welch’s correction. *** p<0.005; **** p< 0.0001
