# Supplementary material for: Cross-linking of T cell to B cell lymphoma by the T cell bispecific antibody CD20-TCB induces IFNγ/CXCL10-dependent peripheral T cell recruitment in humanized murine model
Source: PLoS One. 2021 Jan 6;16(1):e0241091. doi: 10.1371/journal.pone.0241091 (PMC7787458; doi:10.1371/journal.pone.0241091)
Supplement: S5 Fig — a-b) Top: Representative histological staining of WSU DLCL2 tumors 24h post second treatment (0.5 mg/kg CD20-TCB or suitable vehicle i.v.). Bottom: Quantification of total signal intensity/mm2 from histological images of vehicle vs CD20-TCB treatment. Whole slide scans quantification of 4 μm FFPE sections with the software Halo. Statistical analysis: Unpaired 2-tailed t-test with Welch’s correction. p value is shown. (a) Red: IFNγ, Blue: DAPI. Quantification: Total intensity of IFNγ per tumor area. (b) Red: CXCL10, Blue: DAPI. Quantification: Total intensity of CXCL10 per tumor area. c) In the skinfold chamber of NSG mice WSU DLCL2 pretreated or not with IFNγ were injected intradermally together with CD2+ T cells derived from the spleen of HSC-NSG mice and 0.25 mg/kg CD20-TCB or suitable vehicle. Quantification of resident T cell dynamics (Video A-C in S4 Video). Shown are individual track values as scattered dots, and means +/- s.d. Kruskal-Wallis test. ****p<0.0001. d) IFNγ and (e) CXCL10 protein quantification by multiplex analysis of supernant derived from co-culture of WSU DLCL2 cells with decreasing amounts of T cells stimulated with 200 ng/ml of CD20-TCB for 16 hours. Two-way Anova. **p< 0.05, ***p < 0.001, ****p < 0.0001, n.s.: not significant. (PPTX) [file pone.0241091.s005.pptx]

## Slide 1
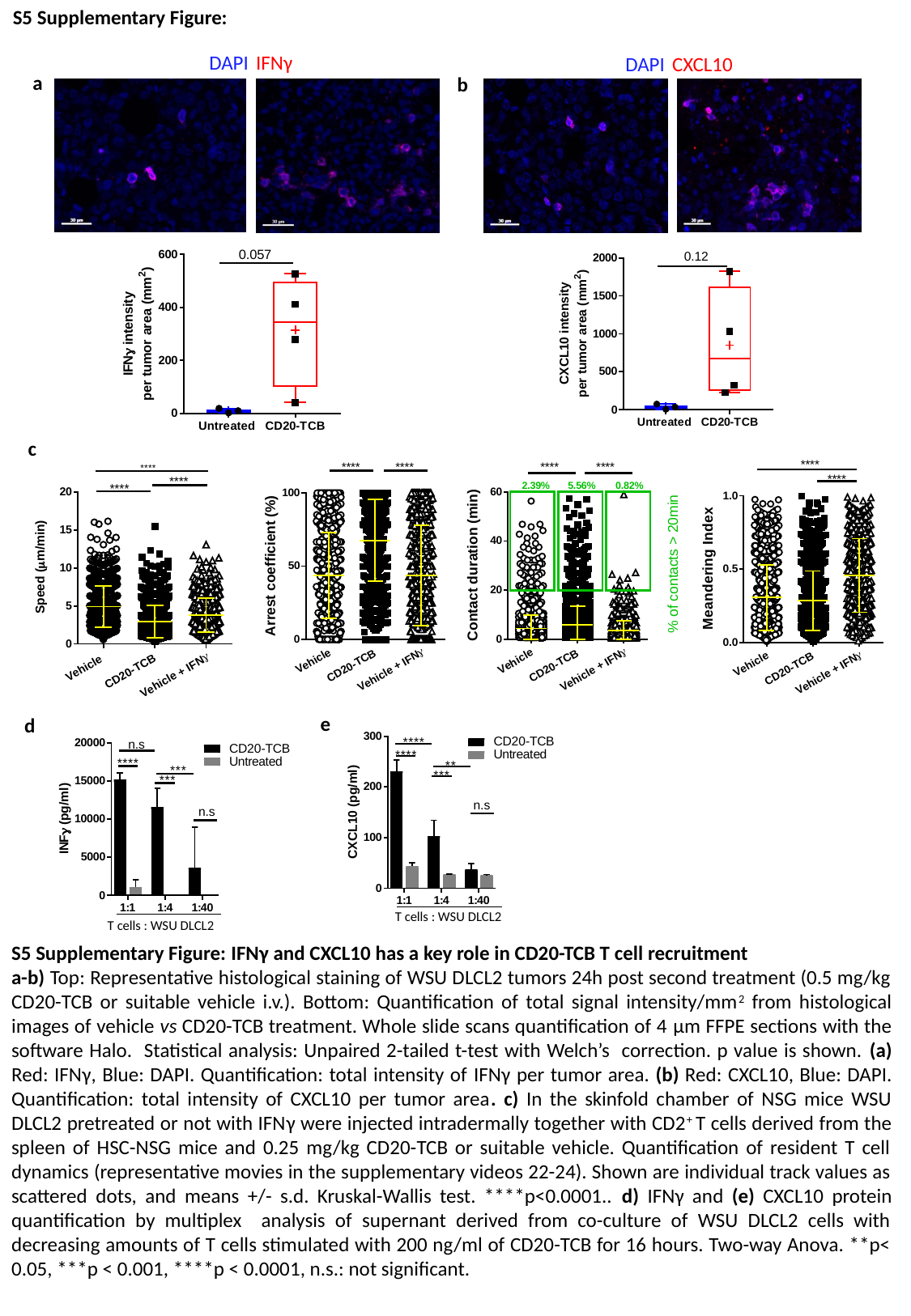

S5 Supplementary Figure:
DAPI
IFNγ
DAPI
CXCL10
a
b
c
e
T cells : WSU DLCL2
d
T cells : WSU DLCL2
S5 Supplementary Figure: IFNγ and CXCL10 has a key role in CD20-TCB T cell recruitment
a-b) Top: Representative histological staining of WSU DLCL2 tumors 24h post second treatment (0.5 mg/kg CD20-TCB or suitable vehicle i.v.). Bottom: Quantification of total signal intensity/mm2 from histological images of vehicle vs CD20-TCB treatment. Whole slide scans quantification of 4 μm FFPE sections with the software Halo. Statistical analysis: Unpaired 2-tailed t-test with Welch’s correction. p value is shown. (a) Red: IFNγ, Blue: DAPI. Quantification: total intensity of IFNγ per tumor area. (b) Red: CXCL10, Blue: DAPI. Quantification: total intensity of CXCL10 per tumor area. c) In the skinfold chamber of NSG mice WSU DLCL2 pretreated or not with IFNγ were injected intradermally together with CD2+ T cells derived from the spleen of HSC-NSG mice and 0.25 mg/kg CD20-TCB or suitable vehicle. Quantification of resident T cell dynamics (representative movies in the supplementary videos 22-24). Shown are individual track values as scattered dots, and means +/- s.d. Kruskal-Wallis test. ****p<0.0001.. d) IFNγ and (e) CXCL10 protein quantification by multiplex analysis of supernant derived from co-culture of WSU DLCL2 cells with decreasing amounts of T cells stimulated with 200 ng/ml of CD20-TCB for 16 hours. Two-way Anova. **p< 0.05, ***p < 0.001, ****p < 0.0001, n.s.: not significant.
