# Supplementary material for: Cross-linking of T cell to B cell lymphoma by the T cell bispecific antibody CD20-TCB induces IFNγ/CXCL10-dependent peripheral T cell recruitment in humanized murine model
Source: PLoS One. 2021 Jan 6;16(1):e0241091. doi: 10.1371/journal.pone.0241091 (PMC7787458; doi:10.1371/journal.pone.0241091)
Supplement: S6 Fig — All surgery steps are performed under a sterile hood in sterile conditions. Surgery preparation: The mouse is anesthetized, appropriate painkiller is injected, and the whole back of the mouse is shaven, and a 1:1 mixture of commercial waxing cream and hydrating cream is applied for 30 seconds to ensure complete shaving. The cream is removed with a wet paper towel (a). Surgery: The back skin of the mouse is then lifted (b) by stitching a surgical thread, to allow the application of the skinfold chamber (c). The desired position of the skinfold chamber is marked, and then one layer of skin is removed to expose the dermis on the opposite side (d). Magnifying the field of view with the help of a stereomicroscope, the hypodermis is carefully removed while maintaining the part moistured (e). The skinfold chamber is then assembled, screws are inserted using a 30G needle to pierce the skin at the assembly points, and the chamber is sewn to the mouse skin using non-resorbable suture thread (f,g,h). The skin of the back is then moisturized with hydrating cream to minimize distress, while painkiller is provided for 48h. Cell injection: 2–3 days post surgery the mice are anesthetized, the coverslip (i) is removed from the skinfold chamber and the cells are injected intradermally in 2 separate injections of 25uL (j) using a curved insulin needle (h) and a stereomicroscope to magnify the area. The window is moisturized and covered again with a coverslip. Imaging can be performed up to 7 days post surgery. Long-term postoperative care requires careful observation of food and water intake, locomotion, behaviour, and signs indicating pain. Research staff will daily examine the surgical site and monitor for signs of infection, incision breakdown, or self-inflicted trauma. (PPTX) [file pone.0241091.s006.pptx]

## Slide 1
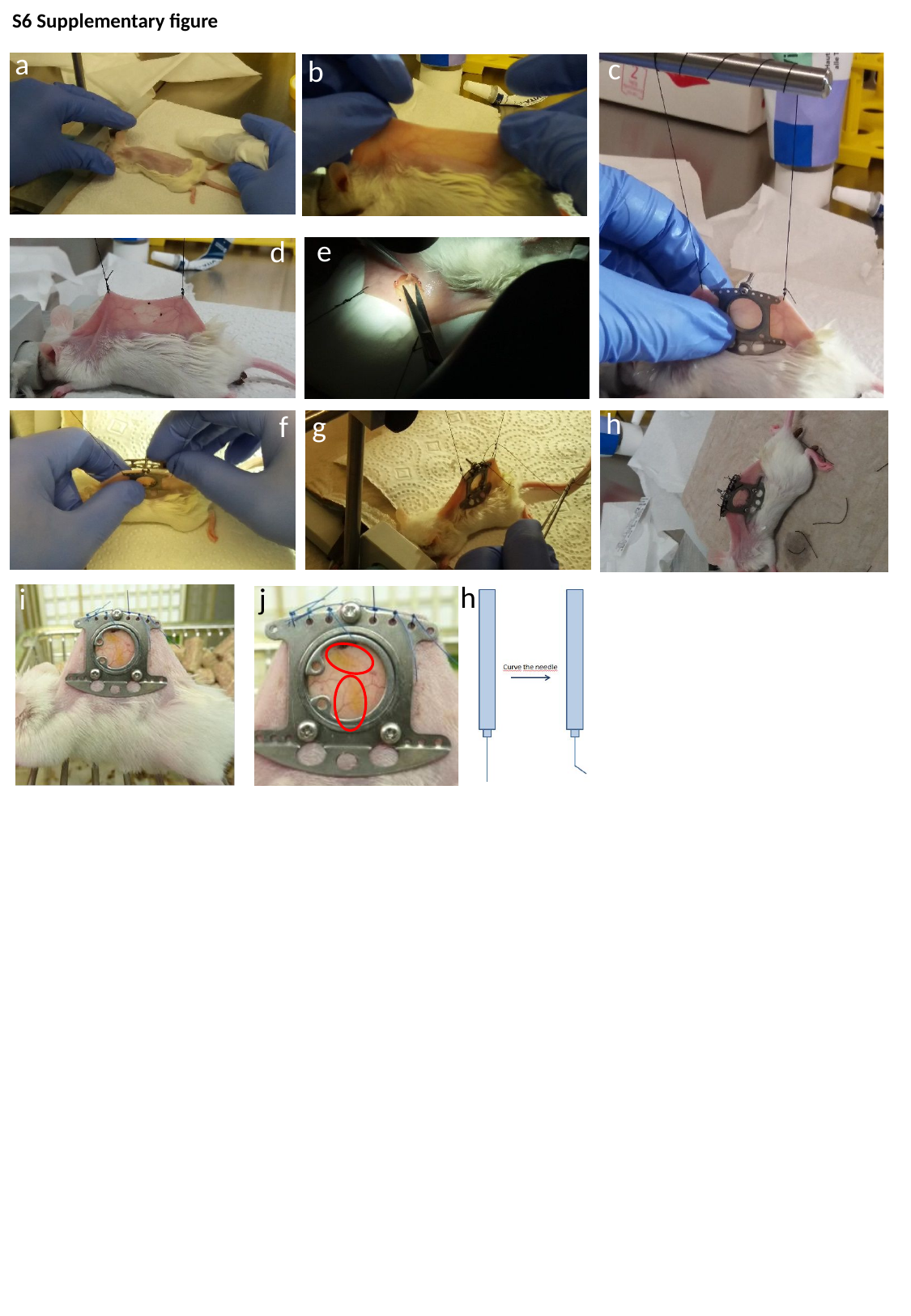

S6 Supplementary figure
a
c
b
e
d
h
f
g
h
i
j

## Slide 2
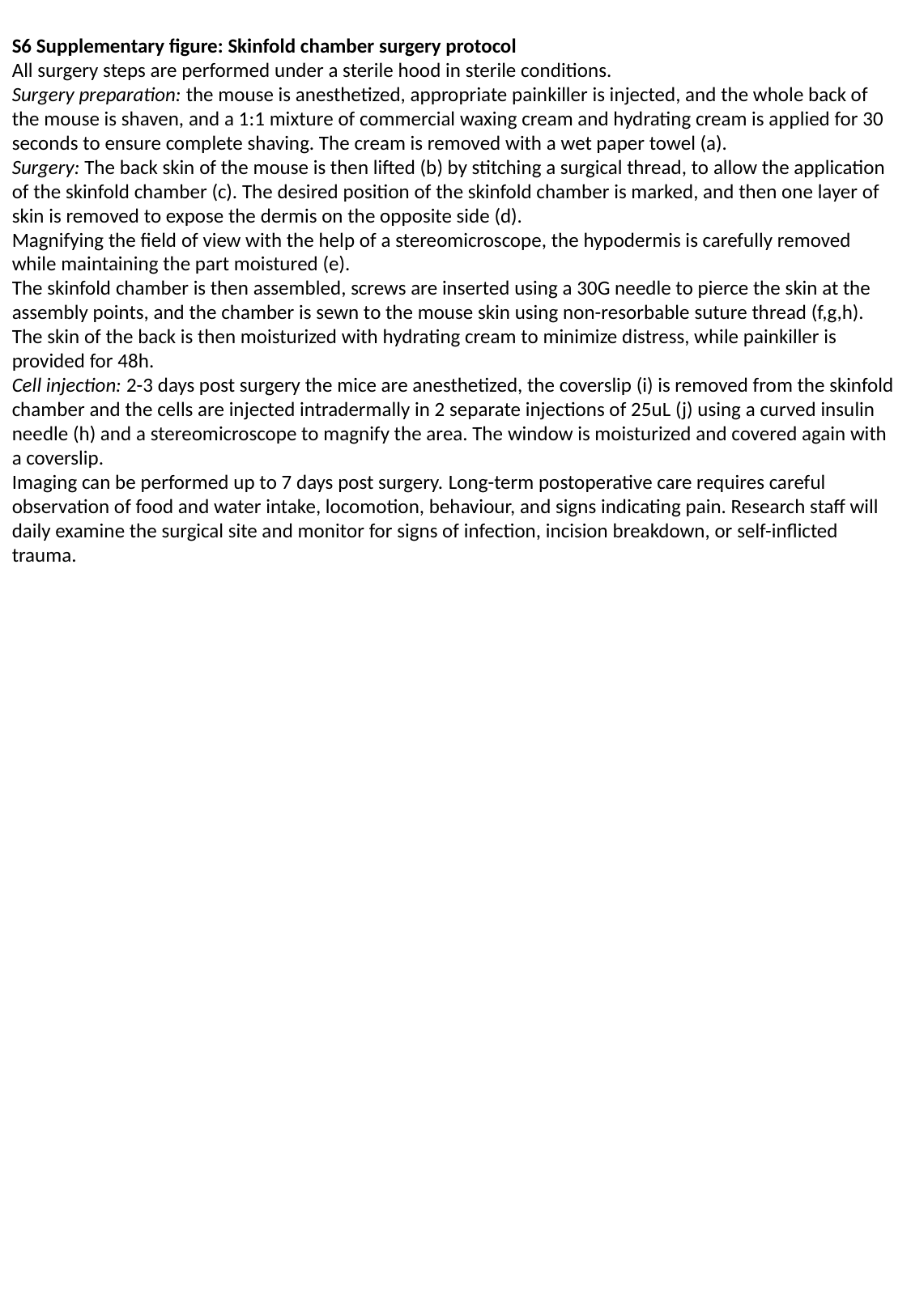

S6 Supplementary figure: Skinfold chamber surgery protocol
All surgery steps are performed under a sterile hood in sterile conditions.
Surgery preparation: the mouse is anesthetized, appropriate painkiller is injected, and the whole back of the mouse is shaven, and a 1:1 mixture of commercial waxing cream and hydrating cream is applied for 30 seconds to ensure complete shaving. The cream is removed with a wet paper towel (a).
Surgery: The back skin of the mouse is then lifted (b) by stitching a surgical thread, to allow the application of the skinfold chamber (c). The desired position of the skinfold chamber is marked, and then one layer of skin is removed to expose the dermis on the opposite side (d).
Magnifying the field of view with the help of a stereomicroscope, the hypodermis is carefully removed while maintaining the part moistured (e).
The skinfold chamber is then assembled, screws are inserted using a 30G needle to pierce the skin at the assembly points, and the chamber is sewn to the mouse skin using non-resorbable suture thread (f,g,h).
The skin of the back is then moisturized with hydrating cream to minimize distress, while painkiller is provided for 48h.
Cell injection: 2-3 days post surgery the mice are anesthetized, the coverslip (i) is removed from the skinfold chamber and the cells are injected intradermally in 2 separate injections of 25uL (j) using a curved insulin needle (h) and a stereomicroscope to magnify the area. The window is moisturized and covered again with a coverslip.
Imaging can be performed up to 7 days post surgery. Long-term postoperative care requires careful observation of food and water intake, locomotion, behaviour, and signs indicating pain. Research staff will daily examine the surgical site and monitor for signs of infection, incision breakdown, or self-inflicted trauma.
