# Supplementary material for: Cross-linking of T cell to B cell lymphoma by the T cell bispecific antibody CD20-TCB induces IFNγ/CXCL10-dependent peripheral T cell recruitment in humanized murine model
Source: PLoS One. 2021 Jan 6;16(1):e0241091. doi: 10.1371/journal.pone.0241091 (PMC7787458; doi:10.1371/journal.pone.0241091)
Supplement: S7 Fig — CD20-TCB by binding to CD3 and CD20 on T cells and tumor cells respectively, allows for the formation of an immunological synapse. Vescicles containing cytotoxic granules (Grazyme B and Perforin) are released in the synaptic area allowing for tumor cell lysis. T cells express LFA-1, that in its active conformation, binds to ICAM-1, on the tumor cell surface, allowing for the stabilization of the immunological synapse. Images adapted from Biorender. (PPTX) [file pone.0241091.s007.pptx]

## Slide 1
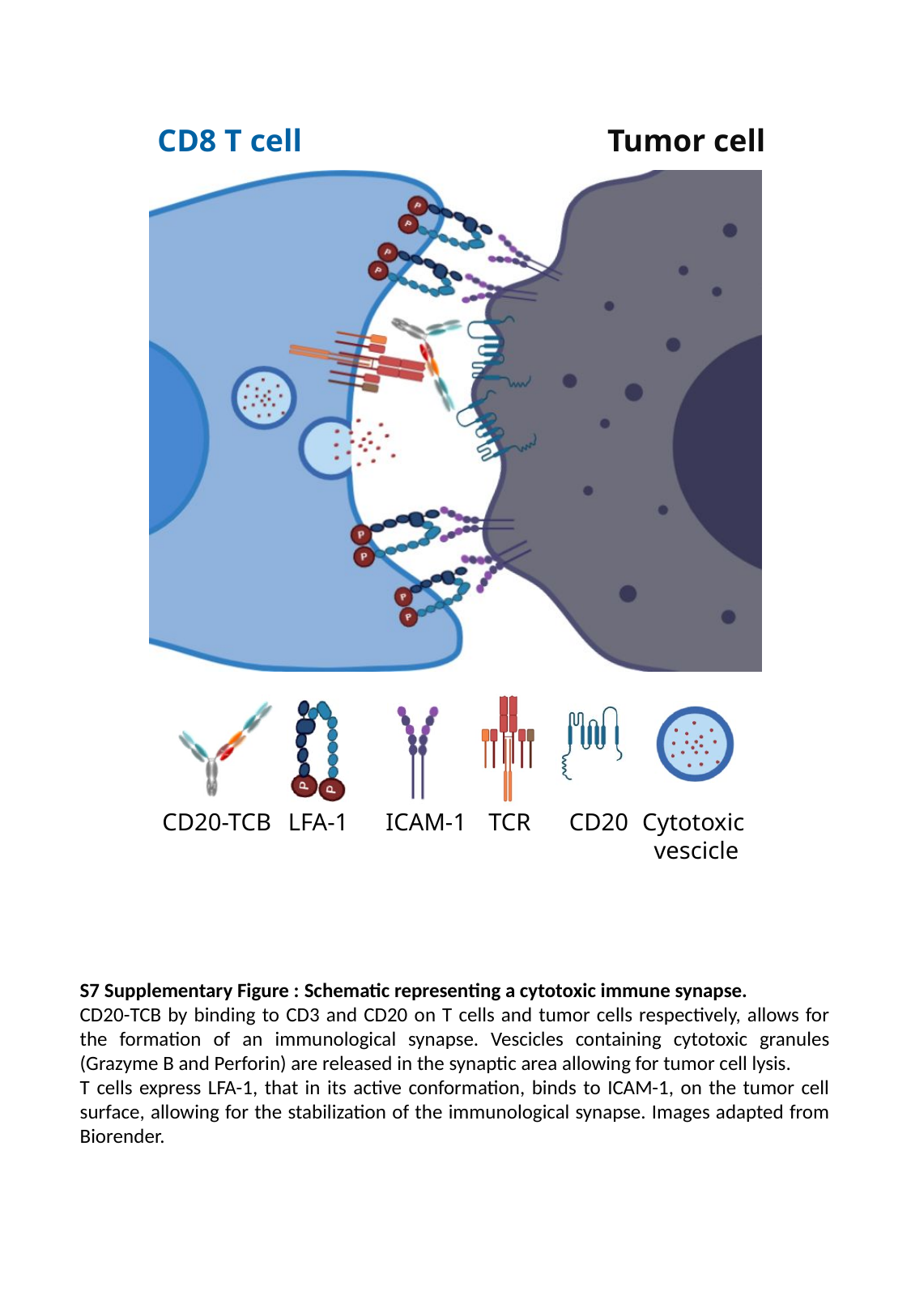

CD8 T cell
Tumor cell
CD20-TCB
LFA-1
ICAM-1
TCR
CD20
Cytotoxic
vescicle
S7 Supplementary Figure : Schematic representing a cytotoxic immune synapse.
CD20-TCB by binding to CD3 and CD20 on T cells and tumor cells respectively, allows for the formation of an immunological synapse. Vescicles containing cytotoxic granules (Grazyme B and Perforin) are released in the synaptic area allowing for tumor cell lysis.
T cells express LFA-1, that in its active conformation, binds to ICAM-1, on the tumor cell surface, allowing for the stabilization of the immunological synapse. Images adapted from Biorender.
