# Supplementary material for: Cross-linking of T cell to B cell lymphoma by the T cell bispecific antibody CD20-TCB induces IFNγ/CXCL10-dependent peripheral T cell recruitment in humanized murine model
Source: PLoS One. 2021 Jan 6;16(1):e0241091. doi: 10.1371/journal.pone.0241091 (PMC7787458; doi:10.1371/journal.pone.0241091)
Supplement: S1 Video — CD8 T cells (pink), tumor cells (blue). (PPTX) [file pone.0241091.s008.pptx]

## Slide 1
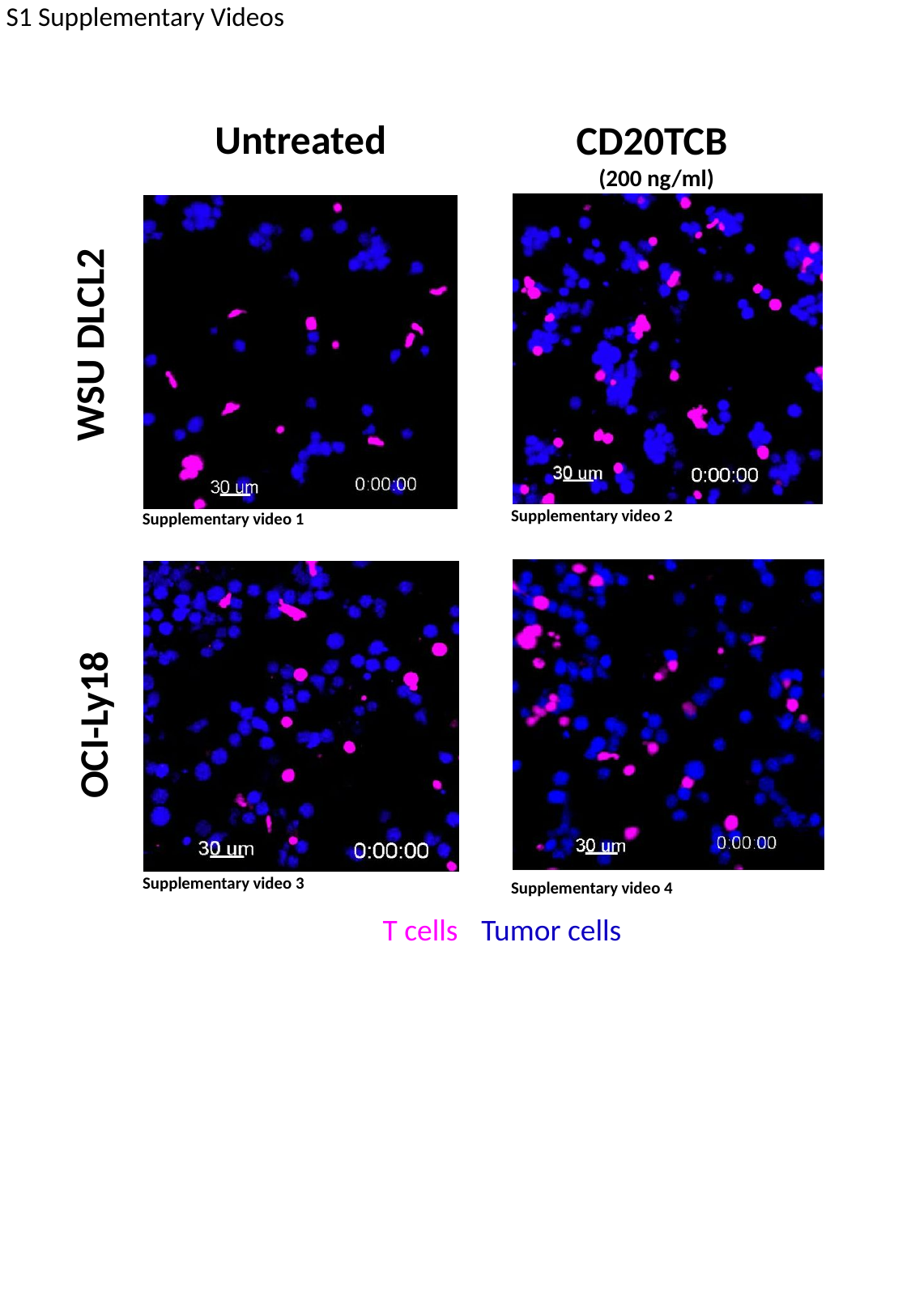

S1 Supplementary Videos
Untreated
CD20TCB
(200 ng/ml)
WSU DLCL2
Supplementary video 2
Supplementary video 1
OCI-Ly18
Supplementary video 3
Supplementary video 4
T cells
Tumor cells
