# Supplementary material for: Cross-linking of T cell to B cell lymphoma by the T cell bispecific antibody CD20-TCB induces IFNγ/CXCL10-dependent peripheral T cell recruitment in humanized murine model
Source: PLoS One. 2021 Jan 6;16(1):e0241091. doi: 10.1371/journal.pone.0241091 (PMC7787458; doi:10.1371/journal.pone.0241091)
Supplement: S2 Video — A, C) vehicle; (B, D) imaged after 2h from CD20-TCB 0.5 mg/kg i.d. injection. Resident CD8 T cells (pink), WSU DLCL2 tumor cells (blue). (PPTX) [file pone.0241091.s009.pptx]

## Slide 1
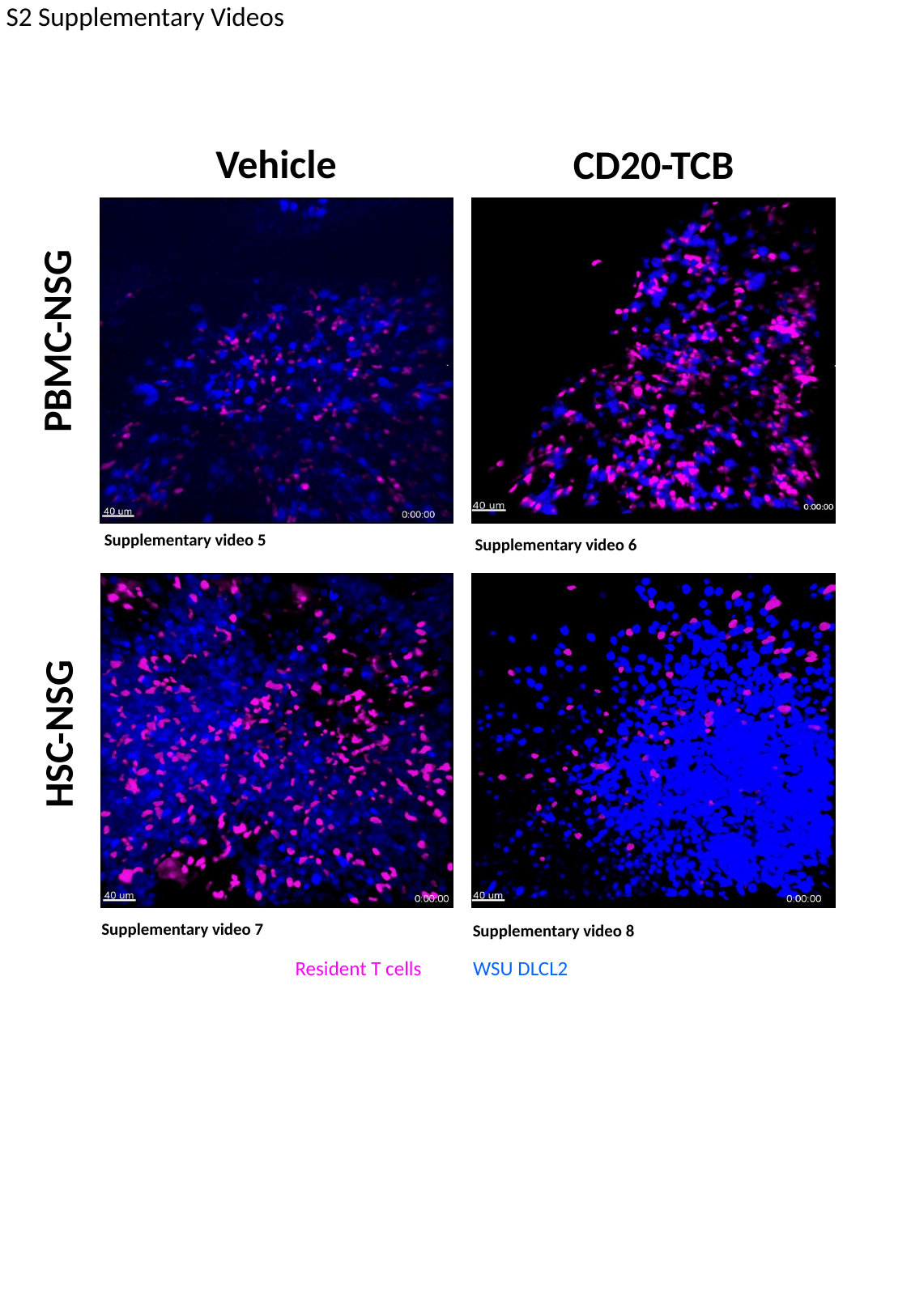

S2 Supplementary Videos
Vehicle
CD20-TCB
PBMC-NSG
Supplementary video 5
Supplementary video 6
HSC-NSG
Supplementary video 7
Supplementary video 8
Resident T cells WSU DLCL2
