# Supplementary material for: Cross-linking of T cell to B cell lymphoma by the T cell bispecific antibody CD20-TCB induces IFNγ/CXCL10-dependent peripheral T cell recruitment in humanized murine model
Source: PLoS One. 2021 Jan 6;16(1):e0241091. doi: 10.1371/journal.pone.0241091 (PMC7787458; doi:10.1371/journal.pone.0241091)
Supplement: S3 Video — Resident CD8 T cells (pink), WSU DLCL2 tumor cells (blue). A-F) Time course imaging of T cell dynamics. A) before injection of Vehicle, B) 0-1h after i.v. injection of vehicle, C) 1-2h after i.v injection of vehicle. D) before, E) 0-1h, F) 1-2h after CD20-TCB (0.5mg/kg) i.v. injection. G) Tracking of T cells after intravenous injection of 0.5 mg/kg CD20-TCB. Highlighted are the 3 different possible behaviors of T cells. i) Light blue track: this T cell is moving fast at the beginning, with a straight trajectory. When it encounters a tumor cell in the presence of therapy, it suddenly stops, and starts interacting. ii) Yellow track: this cell is interacting with the tumor since the beginning, its track is revolving around the same coordinates. iii) Pink track: this cell does not interact with the environment. H-M) Imaging of T cell dynamics at 24h (H), 48h (J) and 72h (L) after i.v injection of vehicle or at 24h (I), 48h (K) and 72h (M) after i.v. injection of CD20-TCB (0.5mg/kg). (PPTX) [file pone.0241091.s010.pptx]

## Slide 1
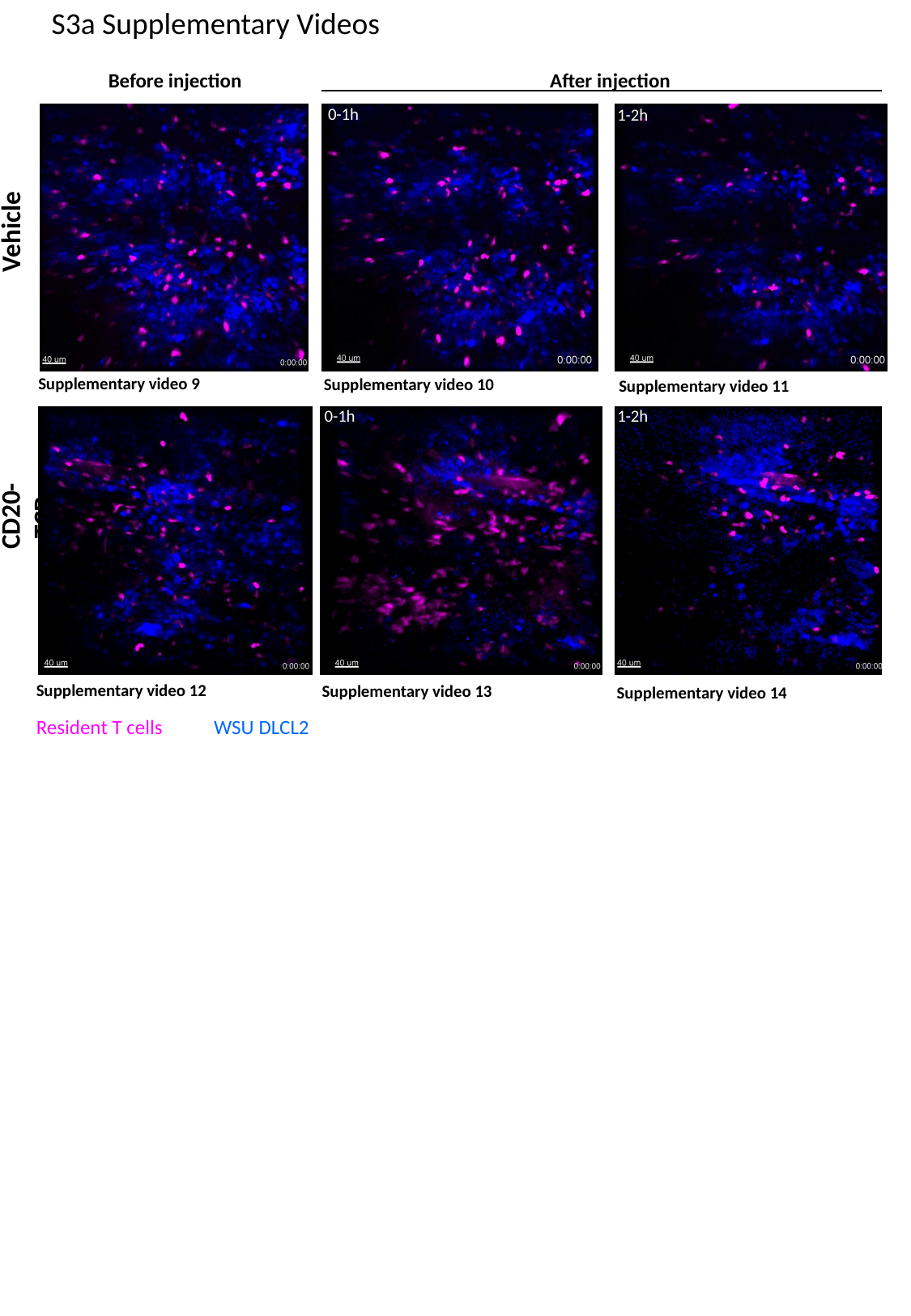

S3a Supplementary Videos
Before injection
After injection
0-1h
1-2h
0-2h
Vehicle
Supplementary video 9
Supplementary video 10
Supplementary video 11
0-1h
1-2h
CD20-TCB
Supplementary video 12
Supplementary video 13
Supplementary video 14
Resident T cells WSU DLCL2

## Slide 2
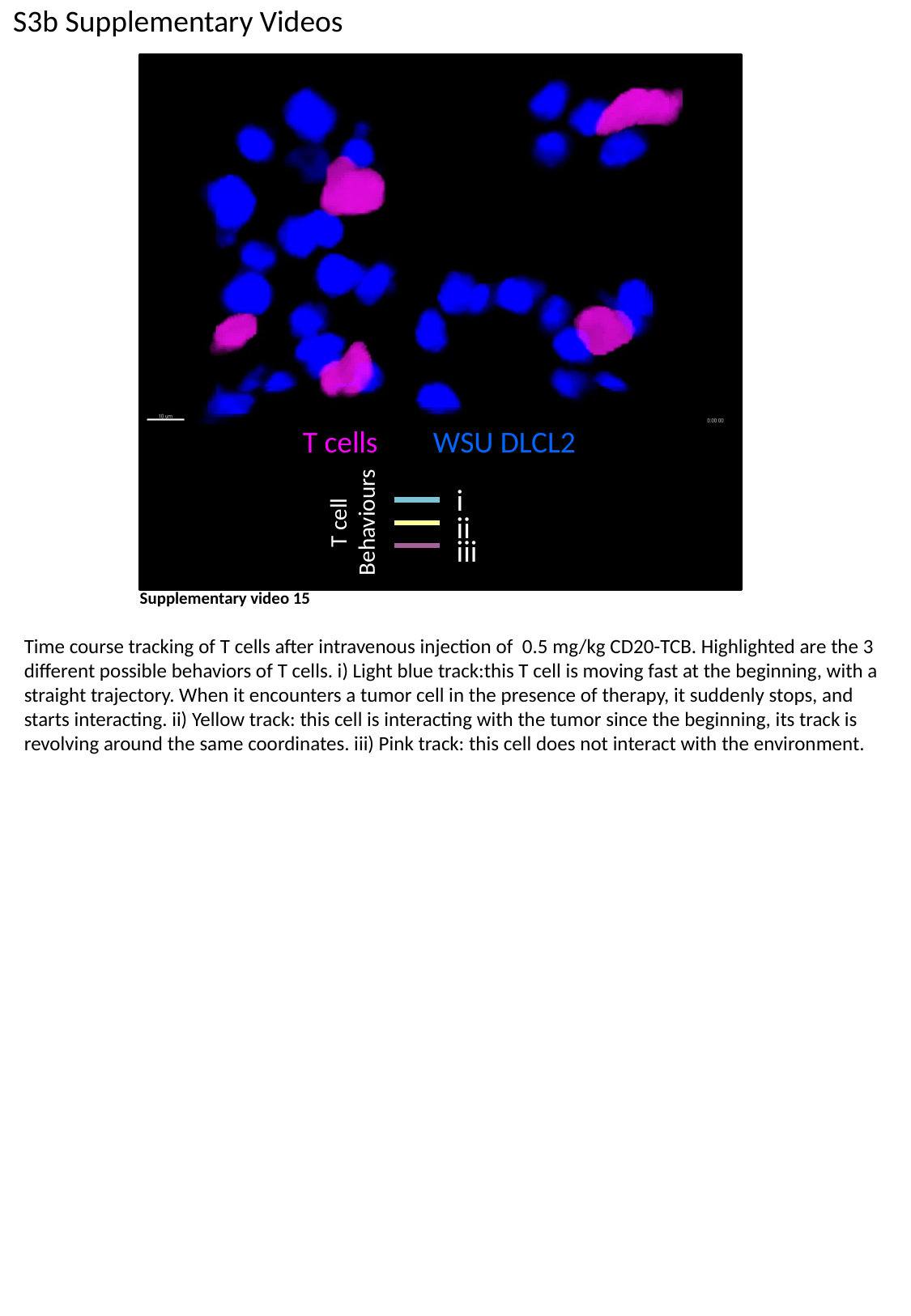

S3b Supplementary Videos
T cells WSU DLCL2
i
T cell
Behaviours
ii
iii
Supplementary video 15
Time course tracking of T cells after intravenous injection of 0.5 mg/kg CD20-TCB. Highlighted are the 3 different possible behaviors of T cells. i) Light blue track:this T cell is moving fast at the beginning, with a straight trajectory. When it encounters a tumor cell in the presence of therapy, it suddenly stops, and starts interacting. ii) Yellow track: this cell is interacting with the tumor since the beginning, its track is revolving around the same coordinates. iii) Pink track: this cell does not interact with the environment.

## Slide 3
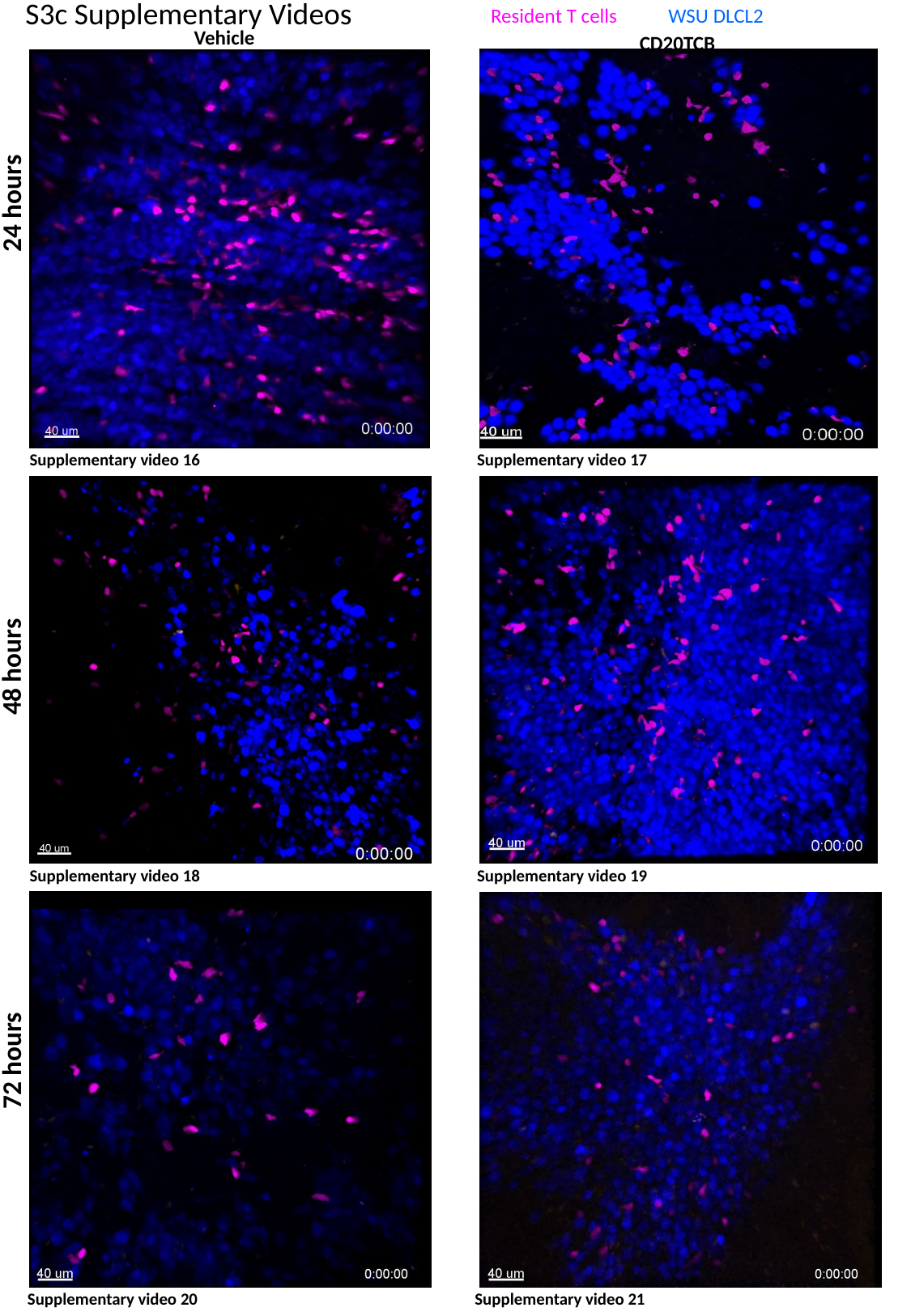

S3c Supplementary Videos
Resident T cells WSU DLCL2
Vehicle
CD20TCB
24 hours
Supplementary video 16
Supplementary video 17
48 hours
Supplementary video 18
Supplementary video 19
72 hours
Supplementary video 20
Supplementary video 21
