# Supplementary material for: Cross-linking of T cell to B cell lymphoma by the T cell bispecific antibody CD20-TCB induces IFNγ/CXCL10-dependent peripheral T cell recruitment in humanized murine model
Source: PLoS One. 2021 Jan 6;16(1):e0241091. doi: 10.1371/journal.pone.0241091 (PMC7787458; doi:10.1371/journal.pone.0241091)
Supplement: S4 Video — Resident T cells (pink), Peripheral T cells (orange), WSU DLCL2 tumor (blue). (PPTX) [file pone.0241091.s011.pptx]

## Slide 1
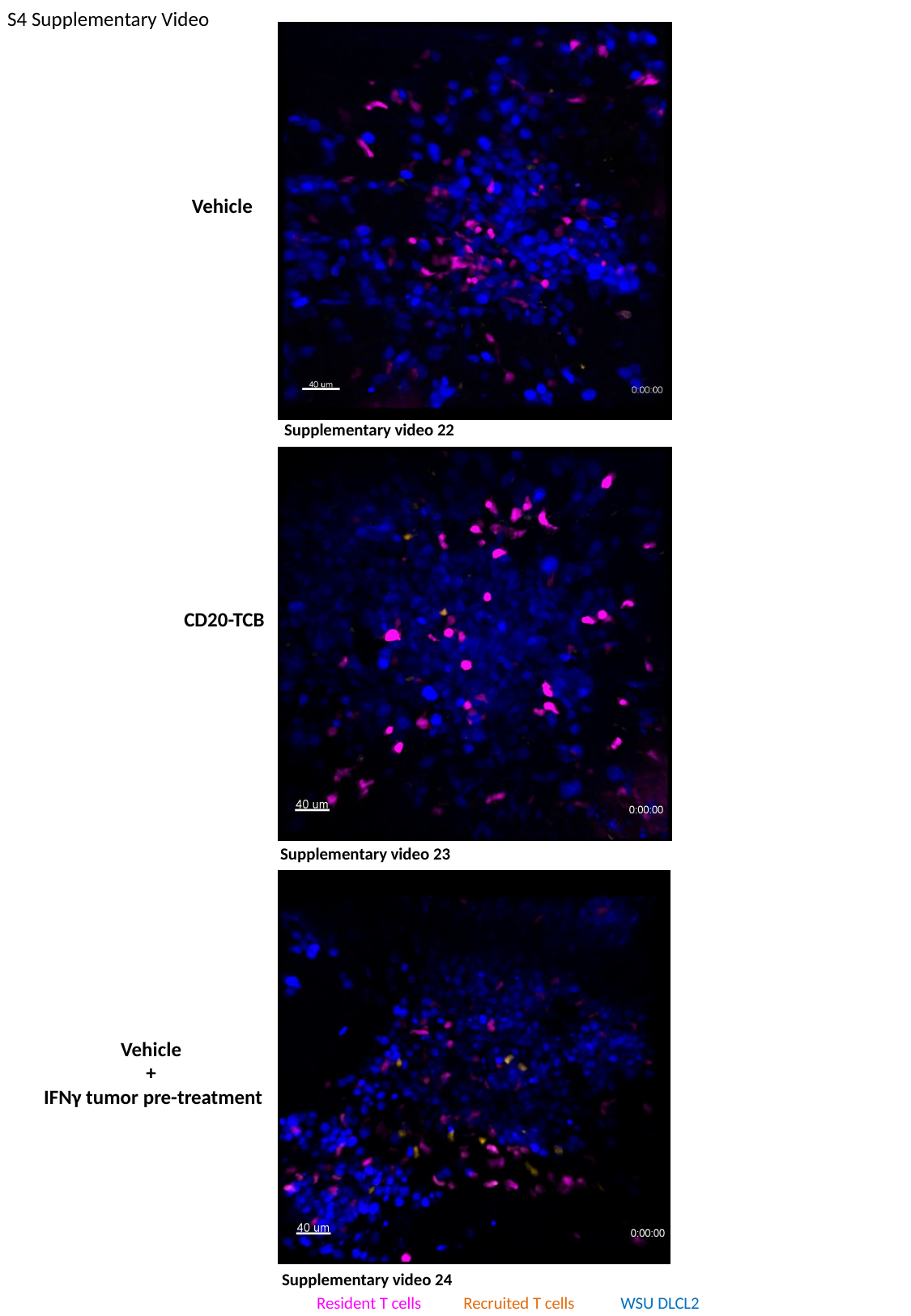

S4 Supplementary Video
Vehicle
Supplementary video 22
CD20-TCB
Supplementary video 23
Vehicle
+
IFNγ tumor pre-treatment
Supplementary video 24
Resident T cells Recruited T cells WSU DLCL2
